# Computational Analysis of G-Quadruplex Forming Sequences across Chromosomes Reveals High Density Patterns Near the Terminal Ends

**DOI:** 10.1371/journal.pone.0165101

**Published:** 2016-10-24

**Authors:** Julia H. Chariker, Donald M. Miller, Eric C. Rouchka

**Affiliations:** 1 Department of Psychological and Brain Sciences, University of Louisville, Louisville, KY, United States of America; 2 Kentucky Biomedical Research Infrastructure Network Bioinformatics Core, University of Louisville, Louisville, KY, United States of America; 3 James Graham Brown Cancer Center, University of Louisville, Louisville, KY, United States of America; 4 Department of Medicine, University of Louisville, Louisville, KY, United States of America; 5 Department of Computer Engineering and Computer Science, University of Louisville, Louisville KY, United States of America; Florida International University, UNITED STATES

## Abstract

G-quadruplex structures (G4) are found throughout the human genome and are known to play a regulatory role in a variety of molecular processes. Structurally, they have many configurations and can form from one or more DNA strands. At the gene level, they regulate gene expression and protein synthesis. In this paper, chromosomal-level patterns of distribution are analyzed on the human genome to identify high-level distribution patterns potentially related to global functional processes. Here we show unique high density banding patterns on individual chromosomes that are highly correlated, appearing in a mirror pattern, across forward and reverse DNA strands. The highest density of G4 sequences occurs within four megabases of one end of most chromosomes and contains G4 motifs that bind with zinc finger proteins. These findings suggest that G4 may play a role in global chromosomal processes such as those found in meiosis.

## Introduction

**Fig 1:**
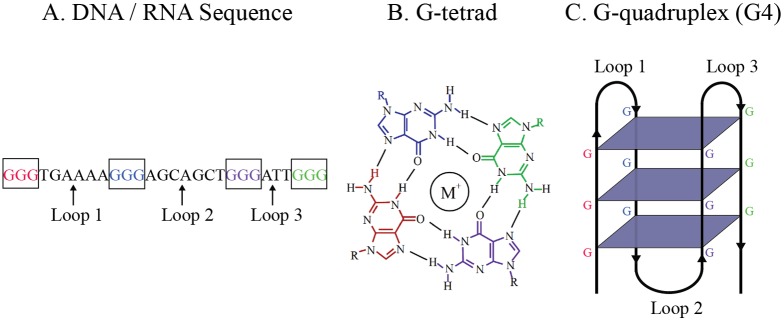
The relationship between (A) a DNA or RNA sequence, (B) a G-tetrad, and (C) the tertiary structure of a G4.

G-quadruplex structures, often referred to as G4, are a form of non-B DNA involved in a variety of molecular processes [1–9]. They are defined by sequences of DNA or RNA that consist of four tracts of guanine bases separated by short (3–7 bp) runs of non-specific nucleotide sequences. The sequence folds to form a three-dimensional structure in which the guanine tracts form stacks of planar structures, known as G-tetrads, while short segments separating the guanine tracts form connecting loops in the three-dimensional structure (see Fig 1). These structures are highly polymorphic, and variations often involve different numbers of G-tetrads and different connecting loop lengths. The configuration that results from the folding process is relevant to the stability and the function of the three-dimensional structure [10].

Sequences capable of forming G4 have been found in telomeres, promoters, introns, exons, 5’ untranslated regions (UTRs), and 3’ UTRs [1, 11–14] with higher associations to genes belonging to particular functional classes such as transcriptional regulation [12, 15, 16]. Experimental evidence points to their role as a regulatory mechanism in processes such as telomerase production [9], gene transcription [3, 4, 6], alternative splicing [17–22], and protein synthesis [2, 5]. More recently, G4 in mRNA have been shown to have a significant role in neurite localization signaling [8], dendrite localization [23], and polyadenylation [1]. Telomeric repeat sequences in humans are capable of forming successive G4 and may serve to protect the telomeric 3’ overhang from degradation [7].

In 2005, Huppert and Balasubramanian used bioinformatics approaches to measure the prevalence of putative G4 sequences on the first assembly of the human genome and found approximately 376,000 sequences capable of forming a G4 structure [24]. Subsequent analyses looked at the distribution of these sequences within specific gene features, such as promoters [12], 5’ UTRs [5, 11], and 3’ UTRs [1], as well as variations on canoncial structure, in particular, structures formed with two, rather than three, G-tetrads [25] and structures formed on both strands of DNA [26]. In general, these studies represent genome-wide analyses of local structural relationships in order to infer the biological functions of G4 at the gene level. However, fundamental biological processes exist at higher scales. One example of this is the positioning of chromosomes and the changes in chromosome condensation across phases of mitosis and meiosis. An analysis of global patterns of G4 distribution may provide insight into their potential role in higher order biological functions.

In this manuscript, visual and computational methods of analysis are used in a complementary fashion to provide a new perspective on the prevalence of putative G4 within chromosomes on the hg38 assembly of the human genome. Although the use of computational methods in the study of G4 is well-known [24], as an analytical tool, visual methods can provide insight into patterns in data that are not easily accessible in numerical or statistical form [27]. As a first step in this analysis, putative G4 were identified using the Quadparser algorithm [24, 28]. Next, using visual and statistical methods, the global distribution of putative G4 was examined across all chromosomes and across DNA strands within individual chromosomes. At a large scale, G4 appear as alternating high and low density bands on all chromosomes. This pattern is strongly similar, nearly a mirror pattern, across forward and reverse DNA strands. On the majority of chromosomes, the highest G4 densities are found within four megabases of the end of the chromosome, a pattern that is distinct from other genomic elements. The potential biological relevance of this pattern is discussed.

## Material and Methods

The reference assemblies and gene annotations for the human genome were downloaded from the UCSC Genome Browser [29] and Ensembl using NCBI build 38, version 78 (GrCh38/hg38) and build 37 (GRCh37/hg19). The starting and ending locations of sequence gaps and chromosome cytobands on the hg38 reference assembly were downloaded from the Mapping and Sequencing section of the UCSC Table Browser for chromosomal assemblies [30]. Experimentally-derived transcription factor binding sites and histone binding sites were obtained from the Ensembl regulation database (*Homo sapiens*, release 84) [31]. A custom Perl program was written to count the guanine (G) and cytosine (C) bases at megabase intervals in hg38 fasta files. Alternate, unknown, and random portions of the hg38 and hg19 assemblies were not considered in this analysis.

Putative G4 were identified using the Quadparser algorithm [24, 28]. Quadparser was set to identify sequences on the forward DNA strand with the pattern G_3+_N_1-7_G_3+_N_1-7_G_3+_N_1-7_G_3+_. Sequences on the reverse DNA strand were identified by substituting C for G in the same pattern. G4 locations identified by high-throughput sequencing (G4-seq) were obtained from the NCBI Gene Expression Omnibus [32] accession GSE63874 [33]. The starting and ending locations of G4 on forward and reverse strands were obtained from bed format files provided for the potassium (K+) G4 stabilization condition.

**Fig 2:**
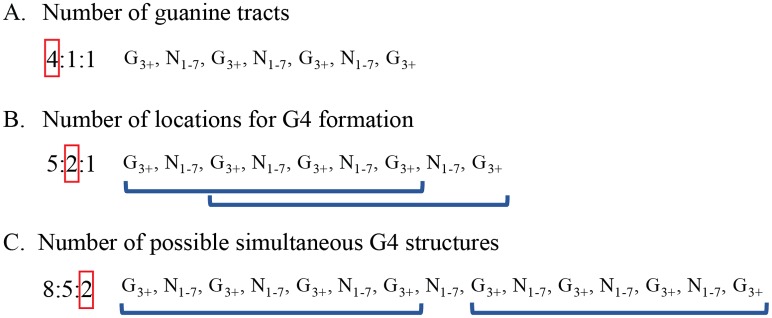
Three types of information available in sequences identified by Quadparser. This information is represented in a sequence code taking the form *x*:*y*:*z*. For each type, example sequences are displayed on the right along with their associated sequence code on the left. Red boxes indicate the position holding that particular information in the sequence code.

Quadparser identified sequences along the genome containing varying numbers of guanine tracts, and this presented a significant challenge for measuring the prevalence of these structures [14, 24]. In Fig 2A, sequences containing four guanine tracts can be counted as a single G4 structure that begins to form at the location of the first guanine tract in the sequence. However, Fig 2B and 2C illustrates the uncertainty in predicting G4 location and composition with sequences containing five or more guanine tracts. In Fig 2B, a single structure may form at multiple locations in the sequence. In this example, with five guanine tracts, a G4 could form at either one of the first two guanine tracts. In Fig 2C, a separate issue arises with sequences containing eight or more guanine tracts. With these sequences, multiple G4 could form simultaneously. To handle these issues, Quadparser codes each sequence in the format *x*:*y*:*z* where *x* represents the number of guanine tracts, *y* represents the number of locations at which a G4 could form, and *z* represents the number of G4 that could form simultaneously in the sequence. The raw data provided by Quadparser, including the sequence, sequence location, and sequence code, is available at http://bioinformatics.louisville.edu/QFS/index.html. S1 Table summarizes the number of G4 identified on each chromosome for different sequence codes.

Several steps were taken to identify high and low G4 density bands along chromosomes. First, a Perl program was written to calculate the number of bases covered by G4 at megabase intervals along all chromosomes. The lengths of G4 sequences were summed to determine the number of bases covered in an interval, and this value was subtracted from the interval size to determine the number of bases not covered by G4 sequences in an interval. Next, a chi-square test was performed to compare the proportion of bases covered by G4 in each interval to the proportion of bases covered by G4 across the rest of the chromosome. The threshold for significance was adjusted using the Bonferroni correction for multiple tests. Specifically, a significance threshold of 0.05 was divided by the number of tests performed on each chromosome (i.e., the number of intervals along the chromosome). Intervals with G4 coverage significantly greater than G4 coverage on the remaining chromosome were consider high density intervals. Intervals with significantly lower G4 coverage than the remaining chromosome were considered low density intervals. Contiguous intervals of high or low G4 density were identified as high and low G4 density bands, respectively.

This method was also used to determine whether the highest density interval on a chromosome had a unique level of G4 coverage. Specifically, chi-square tests with Bonferroni correction were used to compare the proportion of G4 covered in the highest density interval to the proportion of G4 covered in the highest density interval found within a separate band on the same chromosome.

Perl programs were written to analyze the number and distribution of G4 sequences, gene transcripts, experimentally-derived transcription factor binding sites, and histone binding sites at megabase intervals (1,000,000 bp) along chromosomes. These programs have been made publically available on figshare (https://figshare.com/). G4 densities were also calculated as the number of identified sequences within intervals spaced on a log_10_ scale from 1,000 bp to 10,000,000 bp along a chromosome. The distance between G4 was calculated as the number of bases between two adjacent G4 minus the number of bases within any existing assembly gap between the two sequences.

The density of sequences identified by Quadparser and the chromosomal locations of gene transcripts were visualized on ideograms created in Circos, a Perl-based tool for the circular representation of chromosomes and associated data [34]. Although Circos offers a flexible, and visually appealing, approach to displaying patterns of data along chromosomes, in our experience, chromosomes are often mentally represented as linear structures. Therefore, steps were taken to linearize the Circos plots. To accomplish this, each chromosome was initially represented in an individual Circos plot. The length of each chromosome was adjusted to reflect the relative size differences across chromosomes and to maintain a common base scale. As a result, in their circular representation, only the largest chromosome, chromosome 1, formed a complete circle. The circular ideograms were linearized in Photoshop^™^ (Adobe Systems Incorporated, San Jose, CA) using a filtering tool that transforms polar coordinates to linear coordinates [35]. This process produced 24 linear chromosomes of the correct relative lengths. The 24 chromosomes were then assembled into a single image.

The chromosomes were represented in the grayscale pattern typical of a Giemsa stain by providing cytoband boundaries and colors as input files to Circos. The centromeres of chromosomes were represented in a light orange color. Areas along the human genome reference that remain unassembled are represented in black. Gene transcript starting and ending locations and their colors were also provided. Gene transcripts were laid on top of the Giemsa staining pattern, and transcripts from the forward and reverse DNA strands were spatially separated so that transcripts on the forward strand appeared at the top of the linear image in green and transcripts from the reverse strand appeared at the bottom in blue. The density distributions for G4 on each strand were also provided as input files and were located outside the chromosome but directly adjacent to the gene transcripts for their respective strand.

This layout supported a variety of visual analyses. Above all, the density pattern of G4 could be compared across DNA strands in individual chromosomes and across all chromosomes. Furthermore, this density pattern could be compared to the distribution of gene transcripts across different chromatin structures and DNA strands.

TRANSFAC Match was used to identify DNA protein binding sites within one megabase intervals identified as having the highest G4 density on each chromosome [36]. The analysis was performed using TRANSFAC’s vertebrate profile under matrix table release 2016.1 with 4437 matrices. TRANSFAC’s default parameters were used to set cut-offs for core and matrix similarity. A custom Perl program was written to identify DNA protein binding sites overlapping G4 sequences by one or more nucleotides. To a relatively small degree, multiple TRANSFAC matrices identified identical binding sites and this led to duplicate overlaps with G4 in the data. These duplicates were removed prior to analysis.

## Results

### Descriptive Analysis of Identified Sequences

Quadparser identified 197,177 sequences containing four or more guanine tracts along both strands of DNA. The sequences were found in nearly identical proportions on the forward and reverse strands (98,738 and 98,439, respectively). Although the number of guanine tracts within a sequence ranged from 4 to 160, the majority of sequences, 70 percent of the total number of sequences found, contained only four guanine tracts. An additional 20 percent contained five guanine tracts (Table 1).

**Table 1:** The number of putative G4 sequences and the percentage identified with different numbers of guanine tracts.

Number of Guanine Tracts in Sequence	Number of G4 Sequences	Percentage of all Sequences
4	138,907	70.45
5	39,556	20.06
6	11,440	5.80
7	3,817	1.94
8	1,456	0.74
9–160	2,001	1.01
4–160	197,177	100

Nearly 30 percent of the sequences identified contained multiple points at which a G4 could form in the sequence. This resulted in 297,240 locations along the genome capable of structure formation. Again, this number was found in nearly identical proportions on the forward and reverse DNA strands (148,935 and 148,305, respectively).

A vast majority of sequences (over 98 percent of those identified) would allow the formation of only one G4 at any given time (Table 2). Still, there were a large number of sequences remaining across the genome (3,457) that would support the simultaneous formation of 2 to 40 G4, thus providing a foundation for the development of higher-order quadruplex structures [37]. These sequences were found in regions throughout both DNA strands on all chromosomes. The largest number of sequences allowing for higher-order structure were found on chromosomes 1 and 19 (286 each) and the fewest on chromosome Y (20). The number of these sequences per megabase ranged from 0 to 34 with the majority occurring only once per megabase interval.

**Table 2:** The number of putative G4 sequences and the percentage of total sequences identified that would support the formation of one G4 or the simultaneous formation of multiple G4.

Possible Co-occurring G4 in Sequence	Number of G4 Sequences	Percentage of Sequences
1	193,720	98.25
2	2,697	1.37
3–40	760	0.39
1–40	197,177	100

**Fig 3:**
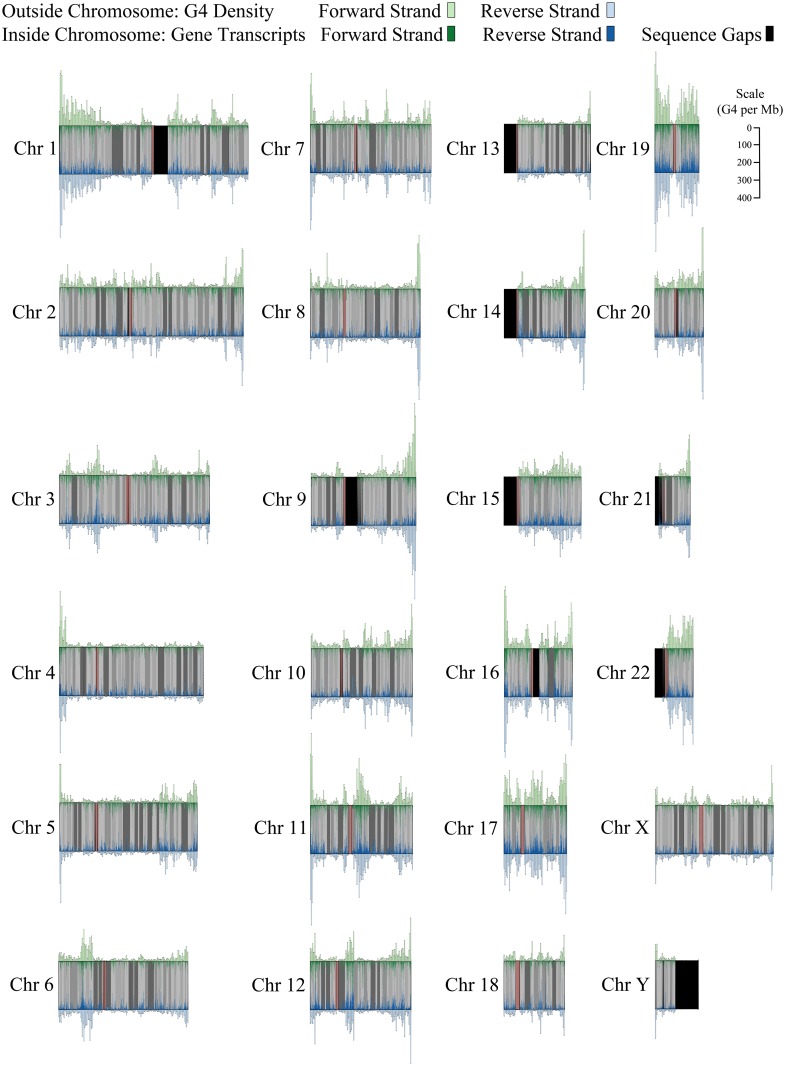
Pattern of G4 sequence density across DNA strands in relation to gene transcripts on each strand. The number of G4 sequences per megabase for the forward strand (green, top) and the reverse strand (blue, bottom) is displayed as a histogram above and below each chromosome. Gene transcripts are displayed inside of each chromosome in corresponding strand colors. Gaps in the reference assembly are represented in black. Centromeres appear in orange.

In an initial visual analysis of G4 distribution, three highly similar global patterns were identified across individual chromosomes (Fig 3). Alternating bands of high and low G4 density appear along the length of each chromosome, and these bands are nearly a mirror pattern across the forward and reverse DNA strands. Most intriguingly, on the vast majority of chromosomes, the highest G4 density is within four megabases of one end of the chromosome. In the following sections, we describe the computational analysis of these patterns.

### Alternating Bands of High and Low G4 Density

In Fig 3, the distribution of putative G4 sequences appears to alternate between bands of high and low density across all chromosomes. This was confirmed by first identifying megabase intervals with G4 coverage significantly higher and lower than G4 coverage on the remaining areas of the chromosome at *p* < 0.01 (see Materials and Methods). The wider high and low density bands were formed from contiguous high and low density megabase intervals along each chromosome. S2 Table displays the number of high and low density bands on each chromosome along with the range of band widths. Not surprisingly, a greater number of bands were found on longer chromosomes. On average, the number of high and low density bands was similar across chromosomes (high: *M* = 11.63, *SD* = 4.6; low: *M* = 12.79, *SD* = 5.33). Nevertheless, when aligning chromosomal end points from the direction of the p arm and the q arm, the width and the density pattern (high,low,or neutral) is unique for each chromosome within three bands.

Fig 3 also illustrates areas that exist along the genome where gene annotations and G4 sequences are conspicuously absent. Many of these are unassembled sections of the genome. Sizeable gaps in the assembly are found on the short arms of several chromosomes and in several sites formed by heterochromatic DNA. Telomeric regions, where the formation of G4 structures is well-known, are also not represented in this assembly. Contigs and scaffolds make up the remaining gaps in the genome. S3 Table provides information regarding the size and types of assembly gaps on each chromosome.

An important question is whether these gaps in the assembly are partially responsible for the variation in G4 sequence density along chromosomes. If this were the case, one would expect to see a negative relationship between the size of unassembled regions and the number of G4 sequences at each megabase interval. However, this relationship was near zero and insignificant for all chromosomes except chromosome X where a significant positive relationship was found (see S4 Table for details). Consequently, there is no reason to believe gaps in the reference assembly play a significant role in the pattern of variation in G4 sequence density along chromosomes.

In Fig 3 it also appears that higher G4 density tends to occur in lighter cytobands where GC content tends to be higher. In fact, it would not be surprising to find high guanine content in areas that are dense with G4 sequences. Guanine tracts are essential to the formation of the guanine tetrads that are fundamental aspects of G4 structure. The Spearman correlations between number of GC nucleotides and number of G4 per cytoband range from 0.44 on chromosome 12 to 0.91 on chromosome 21 with a mean correlation of 0.66 across all chromosomes. All *p* values remain significant after Bonferroni correction with the exception of chromosomes 12, 20, 21, and 24. Clearly, the relationship between the two is not a perfect one, indicating that G4 distribution is only partially related to the changes in GC content across cytobands.

To explore the differences in the two patterns further, we calculated the mean number of G4 per 100,000 bases within different cytoband categories for each chromosome. In Table 3, when looking across all chromosomes, the highest G4 densities occurred in the two lightest cytoband categories (gneg and gpos25) followed by a steep decrease in density, as much as 61 percent from a previous category, over the next four categories (gpos50, gpos75, gpos100, and gvar) as Giemsa staining becomes darker. In contrast, the mean GC content per 100,000 bases also remains high in the two lightest categories but varies little, less than 10 percentage points, through gpos100 with the only large decrease occurring in the gvar category, known to be heterochromatic. If the G4 were simply a by-product of GC content, one would expect the percentage change across the cytoband categories to be fairly similar, but this is not the case, and we see that the difference in the two patterns occurs mainly from gpos50 through gpos100. The mean G4 and GC densities for each cytoband category on individual chromosomes is available in S5 and S6 Tables.

**Table 3:** Mean guanine and cytosine bases (GC) and G4 sequences per 100,000 bases for each cytoband category. The categories gneg (lightest), gpos25, gpos50, gpos75, and gpos100 (darkest) refer to the level of staining achieved. The gvar category refers to areas that tend to be heterochromatic. The acen category refers to centromeric regions. The stalk category refers to the short arm of acrocentric chromosomes.

Cytoband Category	Mean GC per 100,000 bases* (Percentage increase/decrease over previous category)	Mean G4 per 100,000 bases (Percentage increase/decrease over previous category)
gneg	21,094.89	10.74
gpos25	21,538.82 (+2%)	11.33 (+6%)
gpos50	20,175.85 (-6%)	6.89 (-39%)
gpos75	18,244.71 (-10%)	2.71 (-61%)
gpos100	16,982.80 (-7%)	1.52 (-44%)
gvar	4,006.28 (-76%)	1.04 (-32%)
acen	3,776.62	1.07
stalk	1,650.24	1.19

*single strand

### Distribution of G4 Is Highly Similar across DNA Strands

As Fig 3 illustrates, the distribution of G4 is strikingly similar between DNA strands for a given chromosome. Using megabase intervals, G4 density was determined to be highly correlated across strands on all chromosomes, ranging from 0.86 to 0.98 at p < 0.01 with a mean correlation of 0.94 (S7 Table). The G4 strand correlations are significantly higher than gene transcript strand correlations (Mean *r* = 0.65; range 0.43–0.86), indicating a greater similarity in the strand distributions for G4 sequences (S7 Table). It is important to note, however, that this is not a product of complementary structure being identified. G4 sequences that overlap across DNA strands occur rarely on chromosomes, ranging from 0 to 7 instances on individual chromosomes with a mean of 2.29 instances.

**Fig 4:**
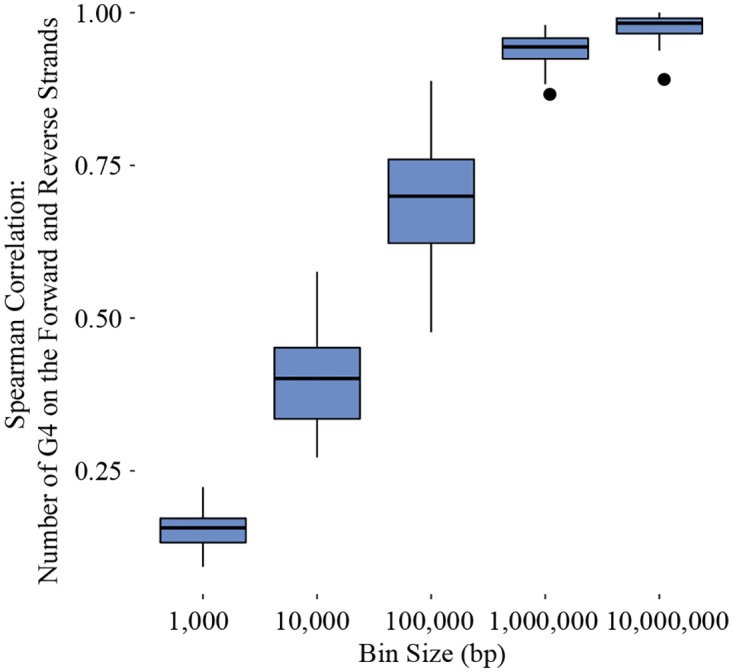
Boxplots representing the range of correlation between the number of G4 sequences on the forward and reverse strands for all chromosomes at different bin sizes. The boxes represent the 25th to 75th percentile, and the end of each line is either the end of the data range or 1.5 of the interquartile range. Dots extending beyond the lines are correlations considered outliers.

**Fig 5:**
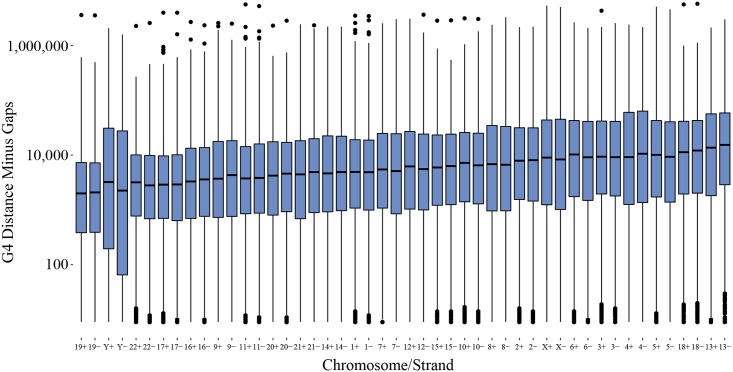
Boxplots representing the range of distances on a log_10_ scale between G4 sequences across all chromosomes and for each strand (represented as a + for the forward strand and a − for the reverse strand). Gaps in the reference assembly are subtracted from all distance measures where they occur. The boxes represent the 25th to 75th percentile, and the end of each line is either the end of the data range or 1.5 of the interquartile range. Dots extending beyond the lines are distances considered outliers for that chromosome.

As can be seen in Fig 4, density patterns across DNA strands increase in similarity at larger scales. The Spearman correlations rise above 0.5 when the number of G4 sequences is measured at intervals above 10 kb (*p* < 0.05 at all intervals). In Fig 5, the median distance and the range of distances between G4 sequences also appears highly similar across DNA strands on individual chromosomes. On a genome-wide scale, G4 sequences are located as near as 9 nucleobases apart and as distant as ~5,800,000 nucleobases with median distances ranging between 1,996 to 15,346 nucleobases. The pattern of G4 sequence distance is in line with the visual pattern of G4 sequence density found in Fig 3, indicating alternating bands of high and low densities exist across chromosomes.

### Highest G4 Density On One End of Most Chromosomes

**Fig 6:**
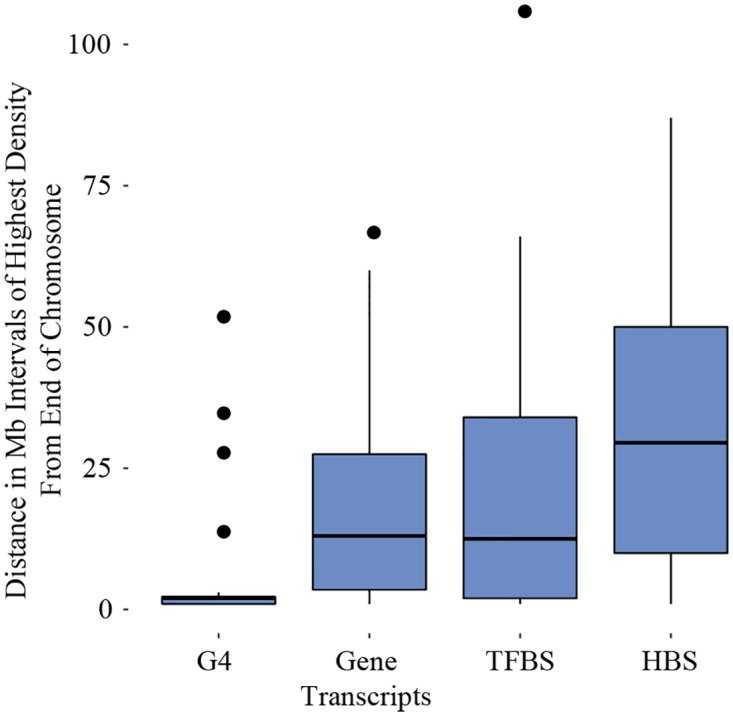
Boxplots representing the range of distances for the highest density megabase interval, measured as the shortest distance to one end of a chromosome, for G4, gene transcripts, transcription factor binding sites (TFBS), and histone binding sites (HBS). The boxes represent the 25th to 75th percentile, and the end of each line is either the end of the data range or 1.5 of the interquartile range. Dots extending beyond the lines are distances considered outliers for that range of distances.

In Fig 3, an interesting pattern can be seen on 21 of 24 chromosomes. In each case, the highest density interval on the chromosome occurs within four megabases of one end of the chromosome. On some chromosomes, this interval has three times as many (or more) G4 sequences than high density intervals found in other bands further along the chromosome. Chromosomes 3, 6, and 15 are exceptions to this pattern. On these chromosomes, the highest density band is found toward the center of the chromosome. At first glance, chromosome 22 appears to be an exception to the pattern as well. However, the number of G4 sequences within one megabase of the end of the q arm is somewhat close to the highest density interval found slightly inward. In S8 Table, the proportion of bases covered by G4 in the highest density interval on each chromosome is signficantly greater than the proportion of bases covered in the highest density interval found among the remaining high density bands on each chromosome (*p* < 0.01), indicating that these intervals are uniquely high on each chromosome. In Fig 6, the pattern of highest density on one end of chromosomes is also distinctive when compared to the pattern of highest density found on gene transcripts, transcription factor binding sites, and histone binding sites (*p* < 0.05).

Within the single megabase region on each chromosome with highest G4 density, binding sites for a wide variety of DNA binding proteins were found. The high density interval on Chromosome Y had the fewest binding sites identified (249,384), the fewest G4 (253), and the fewest binding sites overlapping with G4 sequences (2,511) whereas the high density interval on chromosome 19 demonstrated the highest number of binding sites (329,384), the highest number of G4 (850), and the highest number of binding sites overlapping with G4 (9,319). Information for remaining chromosomes can be found in S9 Table.

**Fig 7:**
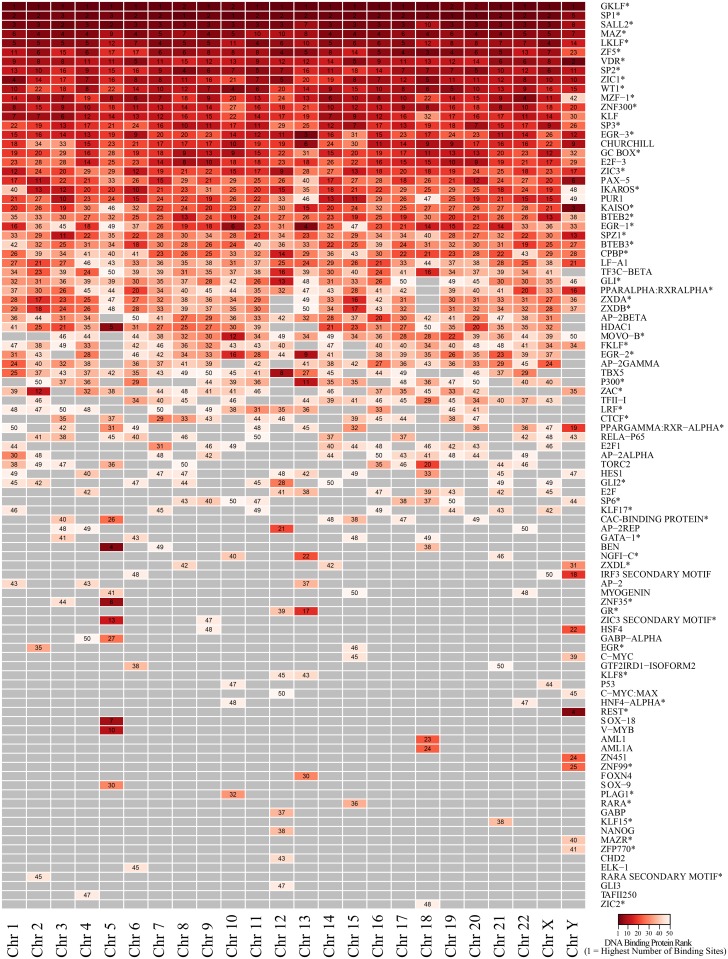
The fifty DNA binding proteins on each chromosome with the greatest number of DNA protein binding sites overlapping G4 sequences. Numbers indicate the rank for a particular DNA binding protein with 1 indicating the highest number of binding sites on a chromosome (darker red) and 50 indicating the lowest number on a chromosome (white).

The fifty DNA binding proteins on each chromosome having the greatest number of binding sites overlapping G4 sequences are listed vertically in Fig 7. On each chromosome, the proteins were ranked so that those with the highest number of binding sites overlapping G4 were ranked 1 (dark red) and those with the fewest were ranked 50 (white). In this group, over half of the DNA binding proteins identified (57 of 99) were zinc finger proteins (indicated by asterisks in Fig 7). This group of zinc fingers is comprised of at least a couple of different structural families with the vast majority being C2H2 zinc fingers and a smaller number consisting of C4 zinc fingers (*VDR*, *PPARα*, *PPARγ*, *HNF4α*, *GR*, *and RARA*). Several zinc finger proteins are among the highest ranked within all chromosomes in terms of number of binding sites overlapping G4. For example, at the top of Fig 7, *GKLF* is ranked number 1 or 2 in abundance across all chromosomes. However, in looking down the figure, many DNA protein binding sites vary in relative abundance across chromosomes, and it is interesting to find that there are also protein binding sites unique to a single chromosome or to a small subset of chromsomes. The raw numbers for DNA protein binding sites overlapping G4 are available in S10 Table.

### Comparison of Predicted to Experimentally Identified G4

The distribution of G4 identified by Quadparser was compared to the distribution of G4 identified experimentally, using a high-throughput sequencing method, G4-seq [33]. The experimentally-derived G4 were identified on the hg19 reference assembly. Therefore, the comparison was made with G4 predicted by Quadparser on the same reference assembly. For each chromosome, Spearman correlations were performed on distributions calculated at one megabase intervals. The distribution of experimentally-derived G4 was highly similar to the distribution of G4 predicted by Quadparser (Mean r = 0.95, range 0.89–0.99, *p* < .01). Strand correlations for experimentally identified G4 were also high (Mean r = 0.91, range 0.74–0.98, *p* < .01), and in S11 Table the strand correlations for the majority of chromosomes show very small differences from strand correlations for G4 predicted by Quadparser. The exceptions are chromosomes X and Y where the strand correlation is higher for predicted G4. The pattern of highest G4 density on one end of most chromosomes is also found with the experimentally-derived data. The highest density interval was the same in both groups with the exception of chromosomes 1 and 20 where the highest density interval differed by 1 megabase but was still located within 3 megabase intervals from the end of the chromosome (S11 Table).

## Discussion

The distribution of G4 across chromosomes is highly distinctive with alternating bands of high and low density that appear in a mirror pattern across DNA strands and highest G4 density located on one end of most chromosomes. Although *a priori*, one might expect a relationship between G-rich structures and alternating GC content across cytobands, the G4 banding patterns were not entirely related to the GC content across different cytobands categories. Nonetheless, just as cytobands serve as a unique signature for identifying chromosomes, the banding patterns of G4 do so as well. In contrast to cytobands, however, the source of the banding is a functional genomic element rather than coloration related to Giemsa staining. Furthermore, this banding pattern is strongly similar across DNA strands showing significantly greater strand similarity than the distribution of gene transcripts across DNA strands. This degree of similarity in strand distributions is a key element in attributing a unique signature to G4, or any element, as one would expect any global chromosomal signature to become increasingly blurred as the distributions of the signature element differ across strands. The pattern of highest G4 density located near one end of most chromosomes distinguishes the distribution of G4 from the distributions of other genomic elements, in this case, gene transcripts, transcription factor binding sites, and histone binding sites, where highest densities on most chromosomes are found at distances that vary more widely and are located further from the ends of chromosomes.

Remarkably, the G4 density patterns identified in this study are in line with a long-standing hypothesis of Sen and Gilbert (1988) regarding a role for these structures in the pairing of homologous maternal and paternal chromosomes (sister chromatids) during meiosis [38]. In 1988, while studying immunoglobulin switch regions, Sen and Gilbert found that guanine-rich regions along DNA self-associated *in vitro*, and on closer analysis, noted that these regions formed parallel four-stranded structures. They proposed that G4 were concentrated in bands at intervals along chromosomes and that they formed a structural component acting like a zipper to align sister chromatids. Sen and Gilbert suggested that the pattern of these bands might act like a “chromosomal fingerprint” for correct homologue pairing.

**Fig 8:**
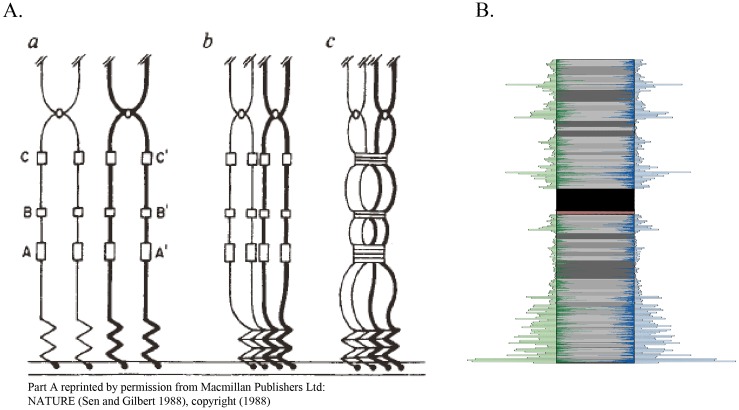
Comparison of Sen and Gilbert (A) to chromosome 1 observations (B). In part A, sister chromatids are attached to the nuclear envelope during meiosis. G-rich bands (A,A’,B,B’,C,C’) serve to align the chromosomes. In part B, highly correlated G4 sequence banding patterns are found along both DNA strands on chromosome 1. Part A reprinted by permission from Macmillan Publishers Ltd: NATURE (Sen and Gilbert 1988), copyright (1988).

In Sen and Gilbert’s original diagram in Fig 8A, the process shows a high density of G4 around the telomeric ends as the sister chromatids attach to the nuclear envelope during meiosis. This fits well in general with the distribution of G4 sequences in this analysis. Exceptionally high densities of G4 sequences are often seen at one or both ends of chromosomes. In addition, bands of guanine-rich sequences along the chromosome are uniquely spaced for each chromosome. In Fig 8B, in addition to the region of highest G4 density near the telomeres, there are approximately five regions with high G4 sequence density. The number, location, and lengths of these bands across chromosomes, together with the high degree of similarity in their distribution across DNA strands, supports the idea that they could serve as a “chromosomal fingerprint”, as Sen and Gilbert suggest, for the correct alignment of chromosomes.

The pattern of highest G4 sequence density occurring within 1 to 4 Mb of one end of 21 out of 24 chromosomes also bears a striking similarity to research on homologue pairing in *Caenorhabditis elegans* [39–43]. Homologue pairing is perhaps best understood in this organism, where specific regions identified on one end of chromosomes, known as pairing centers, are enriched with repeated sequences that bind zinc finger proteins. During meiosis, the zinc finger proteins interact with proteins on the nuclear envelope to facilitate attachment and also serve as a mechanism for linking the pairing centers of homologous chromosomes. As in *C*. *elegans*, we identified regions located on one end of chromosomes containing repeated sequences that bind zinc finger proteins in the current study.

In Sen and Gilbert’s original proposal for homologue pairing, the capacity for interstrand G4 formation was thought to be the basis for alignment and pairing [38, 44], and it is well-known that these self-recognition motifs allow for the formation of G4 from two and four strands of DNA [45]. However, G4 sequence motifs and G4 tertiary structures are also known to bind to a variety of proteins [46, 47]. In fact, several zinc fingers have been significantly associated with G4 motifs in mammals [48]. Perhaps G4 could play a role in homologue pairing through an alternate process, one in which they serve as a set of repeated sequences that bind proteins necessary for correct homologue pairing.

In an alternative scenario for homologue pairing, we now have two factors operating to produce a unique fingerprint for self-recognition, a specific zinc finger binding G4 sequences and the distribution of the G4 sequences within the region. In the current study, it is not obvious that a particular zinc finger would serve as a unique identifier in recognition by itself. Many of the zinc fingers identified in this study bind G4 across all chromosomes. In *C*. *elegans*, although zinc finger proteins critical to homologue pairing do vary across chromosomes, they are not necessarily unique to a chromosome. In this case, it is also noteworthy that the pattern of repeated sequences in *C*. *elegans* differs across chromosomes [39]. Given the findings of our study, along with the research on *C*. *elegans*, it appears that a specific zinc finger distributed in a particular pattern, due to the distribution of G4, is a more likely basis for homologue pairing than zinc fingers operating alone.

Hawley and Gilliland (2009) point out that the pairing center repeats and the telomeric sequences in *C*. *elegans* are highly similar, suggesting a common evolutionary history and implying a possible general mechanism for homologue pairing across species that involves the association of repeated sequences across homologues [39]. Certainly, G4 structures represent a sequence motif with multiple possibilities for binding DNA strands. In the past, this has been most frequently considered in terms of binding strands through the formation of intermolecular G4 structures. However, a variety of proteins, including zinc finger proteins, bind with G4 structures, offering a second mechanism for linking DNA strands. In either of these two cases, the distribution of G4 along the entire length of individual chromosomes, and especially within high G4 density regions, produce unique patterns that could certainly serve as a basis for self-recognition of homologue pairs. The combination of specific zinc finger proteins binding G4 motifs in these regions would increase the precision of any recognition process by using a combination of features to ensure correct homologue pairing.

## Supporting Information

S1 Table Number of G4 sequences identified on each chromosome of the human genome assembly (hg38) by class. (DOCX)

S2 Table The number and range of widths of high and low G4 density bands along each chromosome on reference assembly hg38. (DOCX)

S3 Table The size of unassembled regions within five areas on each chromosome. (DOCX)

S4 Table The proportion of each chromosome that remains unassembled and the correlation between the size of unassembled regions (gaps) per Mb and the number G4 sequences per Mb on each chromosome. (DOCX)

S5 Table The mean number of G4 per 100,000 bases found for each Giemsa staining category for all chromosomes. The categories gneg (lightest), gpos25, gpos50, gpos75, and gpos100 (darkest) refer to the level of staining achieved. The gvar category refers to areas that tend to be heterochromatic. The acen category refers to centromeric regions. The stalk category refers to the short arm of acrocentric chromosomes.
(DOCX)

S6 Table The mean GC content per 100,000 bases found for each Giemsa staining category for all chromosomes. The categories gneg (lightest), gpos25, gpos50, gpos75, and gpos100 (darkest) refer to the level of staining achieved. The gvar category refers to areas that tend to be heterochromatic. The acen category refers to centromeric regions. The stalk category refers to the short arm of acrocentric chromosomes. The number of guanine and cytosine bases were counted on the hg38 reference assembly and thus the values are for one DNA strand. Densities would be the same on the second strand.
(DOCX)

S7 Table Spearman correlations for the distribution of G4 sequences across DNA strands and the distribution of gene transcripts across DNA strands for all chromosomes on reference assembly hg38. The Fisher’s Z estimate and significance of the difference in the G4 strand correlation and the gene transcript correlation is included for each chromosome.
(DOCX)

S8 Table An analysis of intervals with highest G4 coverage along chromosomes. A) Contains information regarding chromosome lengths and number of megabase intervals. B) Indicates the megabase interval with the highest G4 density on each chromosome along with the number of G4 sequences, the number of bases covered by G4 in the interval and the number of bases not covered by G4 in the interval. C) Contains the same information for the highest G4 density interval found in a separate band on the same chromosome. D) Contains the chi square estimate and significance level when comparing the proportion of G4 coverage in B) and C).
(DOCX)

S9 Table The number of DNA protein binding sites, G4 sequences, and DNA protein binding sites overlapping G4 sequences within the megabase interval having highest G4 sequence density on each chromosome. Megabase intervals were measured from the beginning of a chromosome’s P arm. As a result, the last interval listed on the Q arm will be less than 1 megabase in size.
(DOCX)

S10 Table The number of DNA protein binding sites overlapping G4 sequences within the highest G4 sequence density interval (Mb) on each chromosome. The 50 proteins with the highest number of binding sites for each chromosome are included in the table. Protein classes were obtained from TRANSFAC when available. Otherwise, the protein class was obtained from Gene Cards.
(XLSX)

S11 Table Spearman correlations between the distribution (per Mb) of experimentally identified G4 (G4-Seq) and the distribution of computationally predicted G4 (G4-Quadparser) on each chromosome in the hg19 reference assembly. Strand correlations are also displayed for each group along with the the number of megabase intervals on each chromosome and the highest G4 density interval found with G4-Seq and G4-Quadparser.
(DOCX)
