# Supplementary material for: Computational Analysis of G-Quadruplex Forming Sequences across Chromosomes Reveals High Density Patterns Near the Terminal Ends
Source: PLoS One. 2016 Oct 24;11(10):e0165101. doi: 10.1371/journal.pone.0165101 (PMC5077116; doi:10.1371/journal.pone.0165101)
Supplement: S1 Table — (DOCX) [file pone.0165101.s001.docx]

**S1 Table.** Number of G4 sequences identified on each chromosome of the human genome assembly (hg38) by class.

|  |  |  |  |  |  |  |  |  |  |  |  |  |
| --- | --- | --- | --- | --- | --- | --- | --- | --- | --- | --- | --- | --- |
| G4 Class | Chr 1 | Chr 2 | Chr 3 | Chr 4 | Chr 5 | Chr 6 | Chr 7 | Chr 8 | Chr 9 | Chr 10 | Chr 11 | Chr 12 |
| 4:1:1 | 13296 | 9604 | 6309 | 4052 | 5621 | 5618 | 6881 | 5230 | 6713 | 6508 | 8059 | 5688 |
| 5:2:1 | 3847 | 2578 | 1622 | 1126 | 1569 | 1464 | 2024 | 1487 | 1990 | 1804 | 2351 | 1685 |
| 6:3:1 | 1134 | 743 | 460 | 262 | 396 | 417 | 606 | 445 | 556 | 493 | 668 | 509 |
| 7:4:1 | 332 | 202 | 137 | 121 | 124 | 122 | 211 | 151 | 202 | 167 | 239 | 149 |
| 8:5:2 | 121 | 98 | 34 | 31 | 37 | 66 | 65 | 57 | 76 | 82 | 93 | 63 |
| 9:6:2 | 56 | 34 | 13 | 17 | 22 | 29 | 53 | 23 | 37 | 41 | 24 | 24 |
| 10:7:2 | 34 | 19 | 8 | 11 | 9 | 7 | 11 | 15 | 21 | 21 | 23 | 25 |
| 11:8:2 | 27 | 18 | 3 | 7 | 10 | 4 | 10 | 7 | 13 | 8 | 14 | 7 |
| 12:9:3 | 10 | 10 | 1 | 4 | 5 | 6 | 8 | 6 | 6 | 10 | 10 | 5 |
| 13+ | 37 | 30 | 16 | 20 | 20 | 17 | 27 | 26 | 34 | 42 | 34 | 33 |
| Total (Col) | 18894 | 13336 | 8603 | 5651 | 7813 | 7750 | 9896 | 7447 | 9648 | 9176 | 11515 | 8188 |
|  |  |  |  |  |  |  |  |  |  |  |  |  |
|  |  |  |  |  |  |  |  |  |  |  |  |  |
| G4 Class | Chr 13 | Chr 14 | Chr 15 | Chr 16 | Chr 17 | Chr 18 | Chr 19 | Chr 20 | Chr 21 | Chr 22 | Chr X | Chr Y |
| 4:1:1 | 2297 | 4327 | 4908 | 6858 | 8823 | 2517 | 8727 | 4737 | 2002 | 4989 | 4401 | 742 |
| 5:2:1 | 564 | 1158 | 1335 | 2014 | 2634 | 683 | 2940 | 1436 | 530 | 1464 | 1069 | 182 |
| 6:3:1 | 156 | 319 | 327 | 577 | 850 | 188 | 993 | 397 | 166 | 442 | 293 | 43 |
| 7:4:1 | 60 | 122 | 110 | 207 | 290 | 55 | 376 | 122 | 69 | 143 | 83 | 23 |
| 8:5:2 | 24 | 48 | 42 | 79 | 106 | 23 | 130 | 41 | 26 | 71 | 40 | 3 |
| 9:6:2 | 20 | 15 | 10 | 41 | 25 | 16 | 69 | 30 | 13 | 32 | 15 | 5 |
| 10:7:2 | 5 | 16 | 8 | 20 | 25 | 4 | 20 | 12 | 14 | 11 | 5 | 0 |
| 11:8:2 | 6 | 5 | 4 | 7 | 16 | 9 | 24 | 12 | 4 | 13 | 5 | 0 |
| 12:9:3 | 1 | 5 | 1 | 4 | 8 | 2 | 7 | 6 | 5 | 7 | 1 | 1 |
| 13+ | 19 | 12 | 12 | 39 | 46 | 27 | 35 | 22 | 16 | 35 | 21 | 11 |
| Total (Col) | 3152 | 6027 | 6757 | 9846 | 12823 | 3524 | 13321 | 6815 | 2845 | 7207 | 5933 | 1010 |
|  |  |  |  |  |  |  |  |  |  |  |  |  |
