# Supplementary material for: Computational Analysis of G-Quadruplex Forming Sequences across Chromosomes Reveals High Density Patterns Near the Terminal Ends
Source: PLoS One. 2016 Oct 24;11(10):e0165101. doi: 10.1371/journal.pone.0165101 (PMC5077116; doi:10.1371/journal.pone.0165101)
Supplement: S2 Table — (DOCX) [file pone.0165101.s002.docx]

**S2 Table.** The number and range of widths of high and low G4 density bands along each chromosome on reference assembly hg38.

|  |  |  |  |  |
| --- | --- | --- | --- | --- |
| Chromo-some | Chromosome Length  (bp) | Number High Density Bands (range widths in Mb) | Number Low Density Bands (range widths in Mb) | Number of Bands with Neither High nor Low Density (range widths in Mb) |
| 1 | 248,956,422 | 18 (1-13) | 20 (1-54) | 11 (1) |
| 2 | 242,193,529 | 21 (1-13) | 23 (1-39) | 14 (1) |
| 3 | 198,295,559 | 13 (1-12) | 19 (1-37) | 17 (1-2) |
| 4 | 190,214,555 | 15 (1-11) | 19 (1-25) | 14 (1-3) |
| 5 | 181,538,259 | 13 (1-14) | 19 (1-32) | 12 (1) |
| 6 | 170,805,979 | 15 (1-9) | 14 (1-36) | 7 (1-3) |
| 7 | 159,345,973 | 17 (1-5) | 15 (1-25) | 6 (1-2) |
| 8 | 145,138,636 | 12 (1-6) | 18 (1-20) | 11(1-2) |
| 9 | 138,394,717 | 9 (1-13) | 10 (1-33) | 5 (1-2) |
| 10 | 133,797,422 | 17 (1-7) | 16 (1-30) | 7 (1-2) |
| 11 | 135,086,622 | 12 (1-13) | 13 (1-36) | 5 (1) |
| 12 | 133,275,309 | 8 (1-11) | 9 (1-37) | 7 (1-2) |
| 13 | 114,364,328 | 10 (1-14) | 8 (2-22) | 5 (1-3) |
| 14 | 107,043,718 | 6 (1-7) | 8 (1-27) | 4 (1) |
| 15 | 101,991,189 | 17 (1-7) | 16 (1-20) | 9 (1) |
| 16 | 90,338,345 | 9 (1-7) | 9 (2-15) | 3 (1-2) |
| 17 | 83,257,441 | 9 (1-11) | 10 (1-9) | 3 (1) |
| 18 | 80,373,285 | 8 (1-7) | 10 (1-10) | 8 (1-3) |
| 19 | 58,617,616 | 9 (1-6) | 9 (1-13) | 4 (1) |
| 20 | 64,444,167 | 10 (1-4) | 7 (1-19) | 4 (1-2) |
| 21 | 46,709,983 | 5 (1-6) | 5 (1-23) | 1 (1) |
| 22 | 50,818,468 | 4 (1-10) | 5 (1-18) | 3 (1) |
| X | 156,040,895 | 16 (1-6) | 18 (1-26) | 9 (1) |
| Y | 57,227,415 | 6 (1-3) | 7 (1-30) | 4 (1-2) |
