# Supplementary material for: Computational Analysis of G-Quadruplex Forming Sequences across Chromosomes Reveals High Density Patterns Near the Terminal Ends
Source: PLoS One. 2016 Oct 24;11(10):e0165101. doi: 10.1371/journal.pone.0165101 (PMC5077116; doi:10.1371/journal.pone.0165101)
Supplement: S3 Table — (DOCX) [file pone.0165101.s003.docx]

**S3 Table.** The size of unassembled regions within five areas on each chromosome.

|  |  | | |  | |  | |  | |  |
| --- | --- | --- | --- | --- | --- | --- | --- | --- | --- | --- |
| Chromosome | Size (bp) of Unassembled Regions of the Human Genome | | | | | | | |  |  |
|  | Contigs | Hetero- chromatin | Scaffold | | Short Arm | | Telomere | |  |  |
| 1 | 250,500 | 18,000,000 | 199,601 | | 0 | | 20,000 | |  |  |
| 2 | 504,000 | 1,000,000 | 118,388 | | 0 | | 20,000 | |  |  |
| 3 | 150,300 | 0 | 24,915 | | 0 | | 20,000 | |  |  |
| 4 | 330,962 | 0 | 110,626 | | 0 | | 20,000 | |  |  |
| 5 | 153,200 | 0 | 99,081 | | 0 | | 20,000 | |  |  |
| 6 | 600,000 | 0 | 107,255 | | 0 | | 20,000 | |  |  |
| 7 | 200,000 | 50,000 | 105,338 | | 0 | | 20,000 | |  |  |
| 8 | 350,000 | 0 | 0 | | 0 | | 20,000 | |  |  |
| 9 | 1,414,100 | 15,000,000 | 169,964 | | 0 | | 20,000 | |  |  |
| 10 | 150,300 | 0 | 363,716 | | 0 | | 20,000 | |  |  |
| 11 | 224,894 | 207,000 | 100,186 | | 0 | | 20,000 | |  |  |
| 12 | 102,400 | 0 | 14,188 | | 0 | | 20,000 | |  |  |
| 13 | 301,200 | 20,000 | 50,000 | | 15,990,000 | | 20,000 | |  |  |
| 14 | 201,500 | 0 | 264,069 | | 15,990,000 | | 20,000 | |  |  |
| 15 | 150,300 | 0 | 189,261 | | 16,990,000 | | 20,000 | |  |  |
| 16 | 400,300 | 8,000,000 | 111,601 | | 0 | | 20,000 | |  |  |
| 17 | 291,199 | 0 | 25,526 | | 0 | | 20,000 | |  |  |
| 18 | 200,900 | 0 | 61,035 | | 0 | | 20,000 | |  |  |
| 19 | 50,400 | 100,000 | 6,458 | | 0 | | 20,000 | |  |  |
| 20 | 382,149 | 0 | 94,902 | | 0 | | 20,000 | |  |  |
| 21 | 1,501,200 | 50,000 | 50,100 | | 5,000,000 | | 20,000 | |  |  |
| 22 | 1,102,900 | 0 | 35,786 | | 10,500,000 | | 20,000 | |  |  |
| X | 658,011 | 0 | 468,650 | | 0 | | 20,000 | |  |  |
| Y | 549,649 | 30,000,000 | 242,718 | | 0 | | 20,000 | |  |  |
|  |  |  |  | |  | |  | |  |  |
