# Supplementary material for: Computational Analysis of G-Quadruplex Forming Sequences across Chromosomes Reveals High Density Patterns Near the Terminal Ends
Source: PLoS One. 2016 Oct 24;11(10):e0165101. doi: 10.1371/journal.pone.0165101 (PMC5077116; doi:10.1371/journal.pone.0165101)
Supplement: S4 Table — (DOCX) [file pone.0165101.s004.docx]

**S4 Table.** The proportion of each chromosome that remains unassembled and the correlation between the size of unassembled regions (gaps) per Mb and the number G4 sequences per Mb on each chromosome.

|  |  |  |  |
| --- | --- | --- | --- |
| Chromosome | Proportion of Chromosome Unassembled | Pearson *r*:  Gap Size and G4 Sequence Density (per Mb) | Significance  Level (*p*) |
| 1 | 0.074 | -0.05 | 0.471 |
| 2 | 0.007 | -0.05 | 0.439 |
| 3 | 0.001 | -0.09 | 0.210 |
| 4 | 0.002 | 0.06 | 0.400 |
| 5 | 0.001 | -0.01 | 0.850 |
| 6 | 0.004 | -0.04 | 0.580 |
| 7 | 0.002 | -0.06 | 0.469 |
| 8 | 0.003 | 0.04 | 0.628 |
| 9 | 0.120 | -0.05 | 0.542 |
| 10 | 0.004 | -0.01 | 0.883 |
| 11 | 0.004 | 0.02 | 0.850 |
| 12 | 0.001 | -0.04 | 0.652 |
| 13 | 0.143 | -0.06 | 0.548 |
| 14 | 0.154 | -0.06 | 0.549 |
| 15 | 0.170 | -0.10 | 0.299 |
| 16 | 0.094 | -0.09 | 0.419 |
| 17 | 0.004 | 0.06 | 0.575 |
| 18 | 0.004 | -0.03 | 0.803 |
| 19 | 0.003 | 0.00 | 0.975 |
| 20 | 0.008 | -0.03 | 0.801 |
| 21 | 0.142 | -0.09 | 0.556 |
| 22 | 0.229 | -0.16 | 0.250 |
| X | 0.007 | 0.24 | 0.002 |
| Y | 0.538 | 0.01 | 0.948 |
|  |  |  |  |
