# Supplementary material for: Computational Analysis of G-Quadruplex Forming Sequences across Chromosomes Reveals High Density Patterns Near the Terminal Ends
Source: PLoS One. 2016 Oct 24;11(10):e0165101. doi: 10.1371/journal.pone.0165101 (PMC5077116; doi:10.1371/journal.pone.0165101)
Supplement: S5 Table — The categories gneg (lightest), gpos25, gpos50, gpos75, and gpos100 (darkest) refer to the level of staining achieved. The gvar category refers to areas that tend to be heterochromatic. The acen category refers to centromeric regions. The stalk category refers to the short arm of acrocentric chromosomes. (DOCX) [file pone.0165101.s005.docx]

**S5 Table.** The mean number of G4 per 100,000 bases found for each Giemsa staining category for all chromosomes. The categories gneg (lightest), gpos25, gpos50, gpos75, and gpos100 (darkest) refer to the level of staining achieved. The gvar category refers to areas that tend to be heterochromatic. The acen category refers to centromeric regions. The stalk category refers to the short arm of acrocentric chromosomes.

|  |  |  |  |  |  |  |  |  |
| --- | --- | --- | --- | --- | --- | --- | --- | --- |
|  | Mean number of G4 per 100,000 bases | | | | | | | |
| Chromosome | gneg | gpos25 | gpos50 | gpos75 | gpos100 | gvar | acen | stalk |
| 1 | 10.61 | 15.82 | 7.92 | 3.49 | 1.13 | 0.01 | 0.03 | n/a |
| 2 | 7.04 | 5.12 | 5.59 | 2.55 | 1.96 | n/a | 4.67 | n/a |
| 3 | 6.19 | 6.92 | 2.82 | 2.08 | 1.35 | 0.74 | 0.27 | n/a |
| 4 | 4.34 | 3.52 | 1.48 | 1.28 | 0.83 | n/a | 1.5 | n/a |
| 5 | 5.17 | 7.81 | 3.27 | 3.65 | 1.13 | n/a | 0.5 | n/a |
| 6 | 6.08 | 12.06 | 3.27 | 1.83 | 1.24 | n/a | 0.2 | n/a |
| 7 | 9.92 | 7.57 | 3.79 | 2.8 | 1.28 | n/a | 0.15 | n/a |
| 8 | 7.43 | 3.74 | 2.89 | 2.21 | 1.56 | n/a | 0.83 | n/a |
| 9 | 10.31 | 13.52 | 2.62 | 1.89 | 1.59 | 0.14 | 2.12 | n/a |
| 10 | 9.96 | 10.08 | 5.68 | 3.23 | 1.87 | n/a | 0.67 | n/a |
| 11 | 14.75 | 19.85 | 7.9 | 2.77 | 1.23 | n/a | 0 | n/a |
| 12 | 9.04 | 12.71 | 4.45 | 2.23 | 1.43 | n/a | 1.22 | n/a |
| 13 | 3.95 | 5.81 | 2.29 | 0.99 | 1.24 | 0 | 2.54 | 0 |
| 14 | 8.62 | 7.93 | 10.36 | 2.68 | 1.77 | 0.09 | 0 | 0 |
| 15 | 11.04 | 9.56 | 7.71 | 3.23 | n/a | 0 | 2.07 | 0 |
| 16 | 16.48 | 31.12 | 5.68 | 5.04 | 4.9 | 0.42 | 1.63 | n/a |
| 17 | 20.02 | 20.31 | 15.39 | 5.01 | n/a | n/a | 0.46 | n/a |
| 18 | 6.35 | 4.77 | 3.39 | 2.34 | 1.1 | n/a | 0.17 | n/a |
| 19 | 26.02 | 21.67 | n/a | n/a | n/a | 6.54 | 0.55 | n/a |
| 20 | 13.76 | 9.99 | 15.37 | 3.9 | n/a | n/a | 2.26 | n/a |
| 21 | 9.15 | n/a | 5.44 | 1.57 | 0.76 | 2.41 | 0 | 5.97 |
| 22 | 27.5 | 15.93 | 16.74 | n/a | n/a | 1.05 | 2.85 | 0 |
| X | 6.83 | 3.47 | n/a | 2.22 | 1.04 | n/a | n/a | n/a |
| Y | 7.08 | n/a | 17.6 | n/a | n/a | 0.05 | 0 | n/a |
| Mean  (SD) | 10.74 (6.27) | 11.33 (7.09) | 6.89 (5.04) | 2.71 (1.08) | 1.52 (0.90) | 1.04 (1.96) | 1.07 (1.21) | 1.19 (2.67) |
