# Supplementary material for: Computational Analysis of G-Quadruplex Forming Sequences across Chromosomes Reveals High Density Patterns Near the Terminal Ends
Source: PLoS One. 2016 Oct 24;11(10):e0165101. doi: 10.1371/journal.pone.0165101 (PMC5077116; doi:10.1371/journal.pone.0165101)
Supplement: S7 Table — The Fisher’s Z estimate and significance of the difference in the G4 strand correlation and the gene transcript correlation is included for each chromosome. (DOCX) [file pone.0165101.s007.docx]

**S7 Table.** Spearman correlations for the distribution of G4 sequences across DNA strands and the distribution of gene transcripts across DNA strands for all chromosomes on reference assembly hg38. The Fisher’s Z estimate and significance of the difference in the G4 strand correlation and the gene transcript correlation is included for each chromosome.

|  |  |  |  |
| --- | --- | --- | --- |
| Chromosome | G4  Strand Correlation | Gene Transcript Strand Correlation | Fisher's *Z*  (testing significance of difference in *r* values) |
| 1 | 0.96** | 0.71** | 13.02** |
| 2 | 0.93** | 0.55** | 10.73** |
| 3 | 0.88** | 0.58** | 11.30** |
| 4 | 0.86** | 0.55** | 14.89** |
| 5 | 0.91** | 0.64** | 10.63** |
| 6 | 0.92** | 0.52** | 9.18** |
| 7 | 0.94** | 0.60** | 8.10** |
| 8 | 0.88** | 0.43** | 10.68** |
| 9 | 0.96** | 0.58** | 10.18** |
| 10 | 0.95** | 0.50** | 10.52** |
| 11 | 0.96** | 0.73** | 10.78** |
| 12 | 0.94** | 0.63** | 8.70** |
| 13 | 0.94** | 0.73** | 7.72** |
| 14 | 0.96** | 0.75** | 9.38** |
| 15 | 0.95** | 0.72** | 5.87** |
| 16 | 0.95** | 0.80** | 7.92** |
| 17 | 0.96** | 0.74** | 7.22** |
| 18 | 0.92** | 0.52** | 7.75** |
| 19 | 0.97** | 0.59** | 7.74** |
| 20 | 0.93** | 0.71** | 7.73** |
| 21 | 0.97** | 0.86** | 3.87** |
| 22 | 0.98** | 0.82** | 4.87** |
| X | 0.90** | 0.66** | 4.32** |
| Y | 0.95** | 0.56** | 5.04** |
| Mean | 0.94 | 0.65 |  |
| Min | 0.86 | 0.43 |  |
| Max | 0.98 | 0.86 |  |
| **p < .01, Bonferroni correction | | | |
