# Supplementary material for: Computational Analysis of G-Quadruplex Forming Sequences across Chromosomes Reveals High Density Patterns Near the Terminal Ends
Source: PLoS One. 2016 Oct 24;11(10):e0165101. doi: 10.1371/journal.pone.0165101 (PMC5077116; doi:10.1371/journal.pone.0165101)
Supplement: S8 Table — A) Contains information regarding chromosome lengths and number of megabase intervals. B) Indicates the megabase interval with the highest G4 density on each chromosome along with the number of G4 sequences, the number of bases covered by G4 in the interval and the number of bases not covered by G4 in the interval. C) Contains the same information for the highest G4 density interval found in a separate band on the same chromosome. D) Contains the chi square estimate and significance level when comparing the proportion of G4 coverage in B) and C). (DOCX) [file pone.0165101.s008.docx]

**S8 Table.** An analysis of intervals with highest G4 coverage along chromosomes. A) Contains information regarding chromosome lengths and number of megabase intervals. B) Indicates the megabase interval with the highest G4 density on each chromosome along with the number of G4 sequences, the number of bases covered by G4 in the interval and the number of bases not covered by G4 in the interval. C) Contains the same information for the highest G4 density interval found in a separate band on the same chromosome. D) Contains the chi square estimate and significance level when comparing the proportion of G4 coverage in B) and C).

| A) Chromosome Information | | | B) Interval with Highest G4 Coverage | | | | C) Interval with Highest G4 Coverage  in Separate Band | | | | D) *Χ^2^* |
| --- | --- | --- | --- | --- | --- | --- | --- | --- | --- | --- | --- |
| Chromo-some | Chromosome Length (bp) | Number of Intervals | Interval with Highest G4 Coverage | Number G4 Sequences | G4 Coverage (bases) | Uncovered (bases) | Interval with Highest G4 Coverage in Separate Band | Number G4 Sequences in Separate Band | G4 Coverage in Separate Band (bases) | Uncovered in Separate Band (bases) | *Χ^2^* Estimate of Difference in Proportion Covered |
| 1 | 248,956,422 | 249 | 2 | 650 | 19,527 | 980,473 | 157 | 374 | 10,236 | 989,764 | 2943.51** |
| 2 | 242,193,529 | 243 | 241 | 498 | 15,604 | 984,396 | 220 | 309 | 8,927 | 991,073 | 1839.40** |
| 3 | 198,295,559 | 199 | 51 | 332 | 9,094 | 990,906 | 14 | 259 | 7,009 | 992,991 | 271.89** |
| 4 | 190,214,555 | 191 | 2 | 633 | 18,688 | 981,312 | 185 | 68 | 1,733 | 998,267 | 14220.82** |
| 5 | 181,538,259 | 182 | 2 | 506 | 14,928 | 985,072 | 177 | 217 | 6,472 | 993,528 | 3376.65** |
| 6 | 170,805,979 | 171 | 34 | 354 | 10,059 | 989,941 | 42 | 306 | 8,415 | 991,585 | 147.48** |
| 7 | 159,345,973 | 160 | 2 | 551 | 15,490 | 984,510 | 101 | 332 | 9,321 | 990,679 | 1552.62** |
| 8 | 145,138,636 | 146 | 144 | 651 | 19,752 | 980,248 | 23 | 336 | 9,223 | 990,777 | 3881.56** |
| 9 | 138,394,717 | 139 | 137 | 832 | 24,760 | 975,240 | 94 | 222 | 6,143 | 993,857 | 11390.30** |
| 10 | 133,797,422 | 134 | 133 | 469 | 15,471 | 984,529 | 72 | 316 | 8,581 | 991,419 | 1997.17** |
| 11 | 135,086,622 | 136 | 2 | 805 | 24,272 | 975,728 | 65 | 508 | 14,000 | 986,000 | 2810.19** |
| 12 | 133,275,309 | 134 | 133 | 548 | 19,401 | 980,599 | 7 | 348 | 9,889 | 990,111 | 3134.30** |
| 13 | 114,364,328 | 115 | 114 | 334 | 10,969 | 989,031 | 100 | 96 | 2,511 | 997,489 | 5341.70** |
| 14 | 107,043,718 | 108 | 105 | 603 | 17,040 | 982,960 | 25 | 194 | 5,034 | 994,966 | 6601.81** |
| 15 | 101,991,189 | 102 | 75 | 298 | 8,101 | 991,899 | 78 | 274 | 7,469 | 992,531 | 25.77** |
| 16 | 90,338,345 | 91 | 2 | 691 | 19,997 | 980,003 | 89 | 548 | 16,869 | 983,131 | 270.22** |
| 17 | 83,257,441 | 84 | 82 | 623 | 18,089 | 981,911 | 8 | 402 | 11,478 | 988,522 | 1499.91** |
| 18 | 80,373,285 | 81 | 80 | 293 | 9,686 | 990,314 | 49 | 233 | 6,227 | 993,773 | 757.47** |
| 19 | 58,617,616 | 59 | 2 | 850 | 24,821 | 975,179 | 19 | 460 | 13,159 | 986,841 | 3649.58** |
| 20 | 64,444,167 | 65 | 64 | 693 | 20,606 | 979,394 | 58 | 207 | 5,705 | 994,295 | 8550.40** |
| 21 | 46,709,983 | 47 | 46 | 411 | 12,941 | 987,059 | 7 | 147 | 4,114 | 995,886 | 4606.76** |
| 22 | 50,818,468 | 51 | 38 | 452 | 13,005 | 986,995 | 50 | 363 | 10,819 | 989,181 | 202.81** |
| X | 156,040,895 | 157 | 154 | 441 | 12,116 | 987,884 | 1 | 253 | 7,578 | 992,422 | 1055.60** |
| Y | 57,227,415 | 58 | 1 | 253 | 7,578 | 992,422 | 10 | 80 | 2,218 | 997,782 | 2946.12** |
|  | | |  |  |  |  |  |  |  |  |  |

** *p* < .01, Bonferroni correction
