# Supplementary material for: Computational Analysis of G-Quadruplex Forming Sequences across Chromosomes Reveals High Density Patterns Near the Terminal Ends
Source: PLoS One. 2016 Oct 24;11(10):e0165101. doi: 10.1371/journal.pone.0165101 (PMC5077116; doi:10.1371/journal.pone.0165101)
Supplement: S9 Table — Megabase intervals were measured from the beginning of a chromosome’s P arm. As a result, the last interval listed on the Q arm will be less than 1 megabase in size. (DOCX) [file pone.0165101.s009.docx]

**S9 Table.** The number of DNA protein binding sites, G4 sequences, and DNA protein binding sites overlapping G4 sequences within the megabase interval having highest G4 sequence density on each chromosome. Megabase intervals were measured from the beginning of a chromosome’s P arm. As a result, the last interval listed on the Q arm will be less than 1 megabase in size.

|  |  | |  |  |  |  |  |
| --- | --- | --- | --- | --- | --- | --- | --- |
| Chromo-some | | Number Intervals on Chromosome (divided by Mb) | Mb Interval with Highest G4 Sequence Density  (from P arm) | Number DNA Protein Binding Sites (TRANSFAC) | Number G4 Sequences (Quadparser) | Number DNA Protein Binding Sites Overlapping G4 Sequences |  |
| Chr1 | 249 | | 1 | 322,455 | 650 | 6,743 |  |
| Chr2 | 243 | | 240 | 304,729 | 498 | 4,509 |  |
| Chr3 | 199 | | 50 | 292,934 | 332 | 2,781 |  |
| Chr4 | 191 | | 1 | 312,167 | 633 | 6,392 |  |
| Chr5 | 182 | | 1 | 311,816 | 506 | 4,444 |  |
| Chr6 | 171 | | 33 | 311,816 | 354 | 3,169 |  |
| Chr7 | 160 | | 1 | 321,581 | 551 | 5,307 |  |
| Chr8 | 146 | | 143 | 318,675 | 651 | 7,253 |  |
| Chr9 | 139 | | 136 | 321,986 | 832 | 8,925 |  |
| Chr10 | 134 | | 132 | 317,405 | 469 | 5,826 |  |
| Chr11 | 136 | | 1 | 317,290 | 805 | 8,311 |  |
| Chr12 | 134 | | 132 | 311,370 | 548 | 5,798 |  |
| Chr13 | 115 | | 113 | 293,264 | 334 | 3,842 |  |
| Chr14 | 108 | | 104 | 314,248 | 603 | 5,529 |  |
| Chr15 | 102 | | 74 | 284,946 | 298 | 2,534 |  |
| Chr16 | 91 | | 1 | 317,635 | 691 | 6,640 |  |
| Chr17 | 84 | | 81 | 300,276 | 623 | 6,449 |  |
| Chr18 | 81 | | 79 | 312,735 | 293 | 3,108 |  |
| Chr19 | 59 | | 1 | 329,389 | 850 | 9,314 |  |
| Chr20 | 65 | | 63 | 324,375 | 693 | 7,418 |  |
| Chr21 | 47 | | 45 | 315,539 | 411 | 4,454 |  |
| Chr22 | 51 | | 37 | 294,539 | 452 | 4,289 |  |
| ChrX | 157 | | 153 | 306,471 | 441 | 3,940 |  |
| ChrY | 58 | | 0 | 249,384 | 253 | 2,511 |  |
