# Supplementary material for: Computational Analysis of G-Quadruplex Forming Sequences across Chromosomes Reveals High Density Patterns Near the Terminal Ends
Source: PLoS One. 2016 Oct 24;11(10):e0165101. doi: 10.1371/journal.pone.0165101 (PMC5077116; doi:10.1371/journal.pone.0165101)
Supplement: S11 Table — Strand correlations are also displayed for each group along with the the number of megabase intervals on each chromosome and the highest G4 density interval found with G4-Seq and G4-Quadparser. (DOCX) [file pone.0165101.s011.docx]

**S11 Table.** Spearman correlations between the distribution (per Mb) of experimentally identified G4 (G4-Seq) and the distribution of computationally predicted G4 (G4-Quadparser) on each chromosome in the hg19 reference assembly. Strand correlations are also displayed for each group along with the the number of megabase intervals on each chromosome and the highest G4 density interval found with G4-Seq and G4-Quadparser.

|  |  |  |  |  |  |  |
| --- | --- | --- | --- | --- | --- | --- |
| Chromo-some | G4-Seq /  G4-Quadparser  Correlation (hg19) | G4-Seq Strand Correlation | G4-Quadparser  Strand Correlation | Number of Intervals on Chromosome | Highest Density Interval  G4-Seq | Highest Density Interval G4-Quadparser |
| 1 | 0.97** | 0.96** | 0.96** | 250 | 2 | 3 |
| 2 | 0.95** | 0.90** | 0.93** | 244 | 242 | 242 |
| 3 | 0.94** | 0.89** | 0.90** | 199 | 51 | 51 |
| 4 | 0.89** | 0.85** | 0.88** | 192 | 2 | 2 |
| 5 | 0.93** | 0.89** | 0.91** | 181 | 2 | 2 |
| 6 | 0.95** | 0.89** | 0.92** | 172 | 34 | 34 |
| 7 | 0.96** | 0.90** | 0.94** | 160 | 2 | 2 |
| 8 | 0.91** | 0.87** | 0.88** | 147 | 145 | 145 |
| 9 | 0.92** | 0.93** | 0.96** | 142 | 140 | 140 |
| 10 | 0.96** | 0.90** | 0.94** | 136 | 135 | 135 |
| 11 | 0.98** | 0.93** | 0.97** | 136 | 2 | 2 |
| 12 | 0.95** | 0.93** | 0.94** | 134 | 133 | 133 |
| 13 | 0.96** | 0.93** | 0.93** | 116 | 115 | 115 |
| 14 | 0.97** | 0.95** | 0.96** | 108 | 106 | 106 |
| 15 | 0.96** | 0.90** | 0.94** | 103 | 75 | 75 |
| 16 | 0.97** | 0.93** | 0.95** | 91 | 2 | 2 |
| 17 | 0.97** | 0.94** | 0.97** | 82 | 80 | 80 |
| 18 | 0.95** | 0.90** | 0.94** | 79 | 78 | 78 |
| 19 | 0.99** | 0.97** | 0.97** | 60 | 2 | 2 |
| 20 | 0.96** | 0.95** | 0.96** | 64 | 62 | 63 |
| 21 | 0.96** | 0.98** | 0.96** | 49 | 47 | 47 |
| 22 | 0.98** | 0.96** | 0.98** | 52 | 51 | 51 |
| 23 | 0.89** | 0.74** | 0.90** | 156 | 154 | 154 |
| 24 | 0.93** | 0.80** | 0.94** | 60 | 1 | 1 |
| Mean | 0.95 | 0.91 | 0.94 |  |  |  |
| Min | 0.89 | 0.74 | 0.88 |  |  |  |
| Max | 0.99 | 0.98 | 0.98 |  |  |  |

***p* < 0.01, Bonferroni Correction
